# Increasing the Sensitivity of Aspergillus Galactomannan ELISA Using Silver Nanoparticle-Based Surface-Enhanced Raman Spectroscopy

**DOI:** 10.3390/s25144376

**Published:** 2025-07-13

**Authors:** A. D. Vasilyeva, L. V. Yurina, E. G. Evtushenko, E. S. Gavrilina, V. B. Krylov, N. E. Nifantiev, I. N. Kurochkin

**Affiliations:** 1 N. M. Emanuel Institute of Biochemical Physics, Russian Academy of Sciences, 4 Kosygina Str., 119334 Moscow, Russia; alexandra.d.vasilyeva@gmail.com (A.D.V.); lyu.yurina@gmail.com (L.V.Y.); evtushenko@enzyme.chem.msu.ru (E.G.E.); e.gavrilina98@gmail.com (E.S.G.); ikur@sky.chph.ras.ru (I.N.K.); 2 Faculty of Chemistry, Lomonosov Moscow State University, 1/3 Leninskie Gory, 119991 Moscow, Russia; 3 N. D. Zelinsky Institute of Organic Chemistry, Russian Academy of Sciences, 119991 Moscow, Russia; vadimkrilov@yandex.ru

**Keywords:** surface-enhanced Raman scattering, 2,3-diaminophenazine, o-phenylenediamine, ELISA, galactomannan, aspergillosis

## Abstract

Galactomannan (GM) is a polysaccharide secreted by opportunistic pathogenic fungi of the Aspergillus genus. It is prescribed as a diagnostic biomarker of invasive aspergillosis in immunocompromised patients by the guidelines for diagnosis and management of Aspergillus diseases. It has been shown previously that the measurement of soluble horseradish peroxidase (HRP) using surface-enhanced Raman scattering (SERS) of 2,3-diaminophenazine enzymatic reaction product on silver nanoparticles is largely superior in detection limit compared to colorimetric readout. In this study, a highly sensitive SERS-based HRP measurement protocol was applied to enzyme-linked immunosorbent assay (ELISA) for GM quantification in biological fluids. The detection limit for GM was 4.3 pg per sample, which is one and a half orders of magnitude lower compared to colorimetric detection with o-phenylenediamine as a substrate and five times more sensitive than ELISA using 3,3′,5,5′-tetramethylbenzidine.

## 1. Introduction

Invasive aspergillosis (IA) is a high-mortality-rate infection mainly caused by the opportunistic pathogenic fungi belonging to Aspergillus species [1,2,3]. In recent years, cases of invasive aspergillosis have become more frequent among transplant recipients and individuals with HIV. This infection often occurs alongside conditions such as pulmonary tuberculosis, lung cancer, and chronic bronchitis. This has led to IA emerging as a significant cause of infection-related deaths among individuals with weakened immune system [2,4,5].

The spores of Aspergillus are capable of directly invading the airways, resulting in the colonization of the respiratory tract. When disseminated via the bloodstream, they can further infect other internal organs [1,4,6]. Recently, there has been an increase in the number of cases of invasive pulmonary aspergillosis (IPA) among patients with certain viral infections, including COVID-19 and some strains of influenza [7]. Notably, a higher prevalence of IPA has been observed in hospitalized patients with severe COVID-19 who require mechanical ventilation [8].

The early diagnosis of IA is critical for patient survival. However, it is a challenging task due to the absence of specific clinical symptoms [9]. The main component of the cell wall of Aspergillus spp. is galactomannan (GM), a polysaccharide composed of a mannose backbone and a variable number of galactofuranoside side chains [10]. GM detection in serum and bronchoalveolar lavage is recommended by the latest guidelines for the early diagnosis of IA [11,12].

For the detection of GM, the commercial sandwich enzyme-linked immunosorbent assay (ELISA) Platelia™ Aspergillus Ag (Bio-Rad, Marnes-la-Coquette, France) is widely used [12,13,14]. However, false-positive results [15] may arise due to the cross-reactive binding of EB-A2 monoclonal antibodies employed in this assay with certain non-Aspergillus fungi [16,17,18,19,20,21] and bacteria, particularly Bifidobacterium spp., which are essential components of the normal gastrointestinal microbiota in both adults and infants [22,23]. This makes the development of new diagnostic kits with higher sensitivity and specificity important for clinical use.

A previously published paper reported the preparation of highly specific monoclonal antibodies (mAbs) 7B8, capable of recognizing Aspergillus galactomannan. These mAbs were generated using a synthetic immunogen mimicking the natural galactomannan, and their specificity was studied in detail on a glycoarray comprising synthetic oligosaccharides representing distinct structural fragments of Aspergillus GM [24,25,26,27], which were obtained with the use of the pyranoside-into-furanoside rearrangement [28]. Specific binding of new mAbs with *A. fumigatus* and *A. flavus* and the lack of their binding with *Candida albicans*, *Bifidobacterium longum*, *Enterococcus faecalis*, *Escherichia coli*, etc., were confirmed using sandwich ELISA [24]. The obtained results indicate a much higher specificity of the obtained antibodies compared to EB-A2.

The effect of surface-enhanced Raman scattering (SERS) has been widely used in analytical chemistry as a basis for highly sensitive optical assays for various analytes. Considerable efforts have been made to exploit this effect for quantitative measurements of enzymatic activity, especially for horseradish peroxidase (HRP), which is widely used as an enzymatic label in the ELISA. SERS-based measurement of HRP enables more sensitive detection of various antigens compared to classic colorimetry. Several HRP-catalyzed reactions were tested for SERS-based measurements of HRP: oxidation of 3,3′,5,5′-tetramethylbenzidine (TMB) [29] or leuco-dyes [30], with the most sensitive system so far being the oxidative dimerization of o-phenylenediamine (oPD) to 2,3-diaminophenazine (DAP) [31,32]. Recently, an optimized protocol for sensitive SERS-based HRP measurement using this reaction and silver nanoparticles (AgNPs) was published [32], with a limit of detection for HRP of 67 fmol/L (1.3 amol per assay). It describes in detail the procedures for AgNP synthesis and standardization in terms of mean particle size, colloid medium, and particle concentration. It also reports the optimized stop solution for subsequent AgNP aggregation in order to achieve the best conditions for SERS detection of low DAP concentrations in the presence of excess unreacted oPD, as well as procedures for SERS spectra processing. However, this protocol has not been tested in a real immunoassay yet. Even with a well-optimized SERS HRP readout system, the development of new ELISA requires a solution for specific problems, namely the conjugation of chosen antibodies to HRP, the selection of a matrix solution for standard antigen samples, the reduction in signal in blank samples, and its improvement in the presence of antigen.

In the current study, we address all these problems and describe a new SERS-based ELISA for GM which combines the high specificity of 7B8 mAbs with the improved sensitivity of SERS-based readout.

## 2. Materials and Methods

### 2.1. Reagents

All reagents used for the buffers and stop solution preparations are listed in Appendix A. HRP (#P2088, 200–300 pyrogallol units/mg), fetal bovine serum (FBS, #F9665), anhydrous ethylenediaminetetraacetic acid (EDTA, #EDS, ≥99%), and oPD (#P9029, ≥98%) were purchased from Sigma-Aldrich (Burlington, MA, USA). NH_2_OH·HCl (#26103, ≥99%), N-succinimidyl S-acetylthiopropionate (SATP, #26100), and sulfosuccinimidyl 4-[N-maleimidomethyl] cyclohexane-1-carboxylate (Sulfo-SMCC, #22322) were purchased from Thermo Scientific (Waltham, MA, USA). A single-component aqueous TMB substrate solution containing 3,3′,5,5′-tetramethylbenzidine and hydrogen peroxide was purchased from Immunotech (Moscow, Russia). Stabilized H_2_O_2_ 30% *w*/*v* (#141076.1211) was purchased from PanReac AppliChem (Monza, Italy). All other reagents used in the study were >99% purity. An ELISA kit for the detection of galactomannan in a patient’s serum or bronchoalveolar lavage fluid (BALF), GalMAg-ELISA, and anti-galactomannan monoclonal antibody 7B8 were purchased from Xema Ltd. (Moscow, Russia).

### 2.2. Preparing a Culture Medium Containing Galactomannan

*A. fumigatus* was cultivated as a surface culture in a liquid Czapek medium (2 g/L NaNO_3_, 1 g/L KH_2_PO_4_, 0.5 g/L MgSO_4_, 0.5 g/L KCl, 0.01 g/L FeSO_4_, 20 g/L sucrose). The aqueous washout from a two-week culture of *A. fumigatus*, grown in test tubes on slant agar media, was used to inoculate larger volumes of the medium. Subsequently, 10^7^ CFU of fungal spores were added to 150 mL of medium in shake flasks and incubated at 26 °C with stirring for approximately 20 days, until a dense mycelial film with spores formed on the surface of the liquid medium. The fungal growth was carefully removed using a sterile instrument. The remaining liquid was centrifuged at 2000× *g* for 10 min at 4 °C. The supernatant was then treated with acetone for deactivation [24,25,33,34]. The resulting solution served as the analyte for GM determination. GM concentrations in the prepared stock solutions were measured using the GalMAg-ELISA diagnostic test system and its reference standards.

### 2.3. Procedure for Coupling of Antibodies to HRP by Maleimide Method

A solution of mAb 7B8 (0.2 mL, 10 mg/mL) was conjugated with the HRP by the two-step protein crosslinking procedure with SATP and Sulfo-SMCC, according the manufacturers protocol. Briefly, SATP was used for the introduction of protected sulfhydryls into antibodies. Hydroxylamine hydrochloride treatment exposes the labile sulfhydryl group for the conjugation reaction. Sulfo-SMCC, the heterobifunctional crosslinker that contains N-hydroxysuccinimide (NHS) ester and maleimide groups, was coupled with HRP. Coupling reaction between SH-modified mAbs and maleimide-modified HRP was performed at 4 °C under gentle stirring for 8 h. All procedures were carried out in 100 mM Na-phosphate buffer with150 mM NaCl (pH 7.4). Excess crosslinkers were removed by centrifugation on Amicon Ultra Centrifugal filters (UFC5010BK, Merck, Darmstadt, Germany) with a cut-off molecular weight of 10 kDa. Conjugated mAb were stored at 4 °C.

### 2.4. Spiked Samples Preparation

The matrix solution selection details are present in Appendix B. The best chosen option was the supernatant of denaturated human plasma additionally depleted for native GM. A pool of blood plasma was provided by the Moscow Central Station for Blood Transfusion. All participants were healthy and had no prior inflammatory conditions/infections within the last 2 weeks. The study was performed in compliance with the Declaration of ethical principles for medical research involving human subjects.

To prepare the matrix for spike samples, plasma was pre-treated with 0.1 M EDTA and boiled for 3 min to dissociate complexes of polysaccharide with serum components and also to denature proteins. Then, supernatant of the denaturated plasma was prepared by 20 min centrifugation at 14,000× *g*. Native GM was removed from plasma supernatant by magnetic beads modified by mAb 7B8. Known amounts of stock concentrations of GM in 100 mM Tris-HCl, 150 mM NaCl, pH 8 containing 0.05% Tween-20 (TBST) were spiked to the prepared matrix solution in a 1:9 ratio to avoid significant dilution.

### 2.5. Colorimetric ELISA on mAbs Coated Plate

The wells of a 96-well clear polystyrene high-bind stripwell microplate (# 2592, Corning, NY, USA) were filled with mAb 7B8 (100 μL/well of a 10 μg/mL solution in 100 mM Tris-HCl, 150 mM NaCl, pH 8) and incubated for 16 h at 4 °C. After washing three times with TBST, the plate was blocked by 1% BSA solution in TBST (16 h at 4 °C). After blocking, 100 μL HRP-conjugated mAb 7B8 (10 µg/mL in TBST, see Appendix B for selection) and 100 μL of GM-spiked solution were added into the wells and incubated for 60 min at 37 °C. After washing five times, enzymatic reaction was started by the addition of 100 μL of either single-component TMB or oPD substrate mixture, containing 1 mM oPD and 80 µM H_2_O_2_ in 100 mM citrate buffer at pH 6 with 5 µg/mL of BSA. Enzymatic oxidation of TMB or oPD was performed for 15 min at 37 °C with 600 rpm stirring and stopped with 100 μL of 0.5 M sulfuric acid or 200 μL of 1.5 M citrate buffer (pH 3) for TMB or oPD, correspondingly. Absorbance was measured at 450 or 454 nm, respectively, using a xMark plate reader (BioRad, Hercules, CA, USA). Results were represented as means ± standard deviation (SD).

### 2.6. Method of AgNPs Synthesis

AgNPs were synthesized using the modified hydroxylamine method by Leopold and Lendl [35], as previously described [32,36]. To ensure repeatable mean particle size, colloid medium, and particle concentration, selection and standardization procedures were performed for each synthesized batch of AgNPs according to protocol [32]. All details of these procedures are provided in Supplementary Materials.

### 2.7. SERS-Based ELISA

All immunochemical steps were performed as described above, including addition of the same oPD substrate mixture (1 mM oPD and 80 µM H_2_O_2_ in 100 mM citrate buffer at pH 6 with 5 µg/mL of BSA). Instead of stopping the enzymatic oxidation of oPD in a microplate well, an aliquot of reaction mixture (20 µL) was transferred to a tube, where the reaction was stopped with a double volume of 1.5 M citrate buffer, pH 3. All further procedures (addition of AgNPs, spectra acquisition, and their processing) were performed as described previously [32]. Full details of SERS-based ELISA readout are provided in Supplementary Materials. Briefly, the solution was mixed with standardized AgNPs in 1:1 ratio, and two minutes later the SERS spectra were collected from the suspension of aggregated AgNPs using the portable spectrometer i-Raman Pro BWS475–785 H (BWTek, Plainsboro, NJ, USA) with a 785 nm excitation and 20× objective. Spectra were processed according to previously reported recommendations. The results were represented as means ± SD. For presentation purposes, the polynomial baseline was subtracted from each spectrum using OPUS 7.0 software (Bruker Optik GmbH, Ettlingen, Germany).

### 2.8. Processing of Calibration Curves

Each sample was assayed in triplicate within the same plate and the experiment was repeated three times on different days to assess the variance between runs of sample replicates on different plates. Data processing of all calibration curves was carried out in Microsoft Excel 2016. The limit of detection (LOD) was calculated from the calibration curve as the concentration, corresponding to the signal equal to the mean of the blank + 3 standard deviations of the blank. As long as the standard deviation typically strongly fluctuates for small sample sizes (N), the mean coefficient of variation (CV) for the range above the LOD was used as an estimator of assay repeatability. For each concentration, the signal SD was calculated and normalized by the difference between the mean signal and mean blank. The resulting CVs were averaged for all concentrations above the LOD.

## 3. Results and Discussion

The results of numerous studies, generalized and systematized in [37], indicate that baseline and trends in serum GM kinetics correlate with outcome (both response to therapy and survival) in IA, which is very important given the high prevalence of this disease in immunocompromised patients and its high mortality in the absence of timely treatment.

Existing methods for galactomannan detection mainly include ELISA [34] and latex agglutination tests [38]. The commercially available sandwich ELISA (Platelia™ Aspergillus; Bio-Rad) is able to detect GM at concentrations as low as 0.5 to 1 ng/mL in serum [34], in accordance with the EORTC/MSG disease classification system [38]. The results are reported as optical density index (ODI), which is calculated as the ratio of the absorbance of the clinical sample to the manufacturer’s reference controls (the cut-off controls). Since GM is a water-soluble carbohydrate, samples of fluids such as urine, cerebrospinal fluid, pleural fluid, and bronchoalveolar lavage from patients with invasive aspergillosis can be used to detect GM as well [39,40,41]. The analysis of urine samples from patients is promising as it is non-invasive, and the detection of GM in these samples reveals that at least some galactomannan is excreted through the kidneys. However, our understanding of the pharmacokinetics of GM, its renal excretion, and the correlation between urinary GM detection and disease progression remains incomplete. Additionally, the presence of GM antibodies in serum may reduce renal excretion of the antigen, leading to lower urinary GM concentrations. Further studies are needed to explore GM kinetics and elimination during infection across different patient groups, requiring highly sensitive and selective quantitative methods. Such research would also help to determine the dynamics of GM during antifungal therapy and its behavior in various fluid samples.

Improved specificity could be achieved with previously reported 7B8 mAbs [24,27,28]. The solution for the problem of insufficient sensitivity is the usage of a different readout technique, for instance SERS instead of colorimetry. Recently, a new protocol for the SERS-based detection of a HRP enzymatic label based on oxidative dimerization of oPD into DAP was described. The silver nanoparticles’ synthesis and standardization protocol as well as conditions for SERS procedures were previously extensively evaluated and optimized for detection sensitivity [32]. In the present paper, we applied this protocol to the Aspergillus GM ELISA. Since the GM is a multivalent antigen, the typical sandwich GM ELISA design employs identical antibodies both for capture and detection. We followed the same design; 7B8 mAbs were immobilized on the plate surface, and the same 7B8 mAbs were conjugated with HRP using maleimide protocol.

SERS-based readout enables the measurement of lower HRP quantities compared to the colorimetric method. In order to exploit this advantage for the HRP label in ELISA, it is highly important to have as low a HRP label at zero antigen concentration as possible. The two main causes of peroxidase activity in blank samples are non-specific conjugate sorption and the presence of minor amounts of native GM in the plasma of healthy donors. To solve this problem, the optimal concentration of HRP-conjugated mAb 7B8 and the composition of the solution for GM-spiked samples [42,43] were selected (Appendix B).

**Figure 1:**
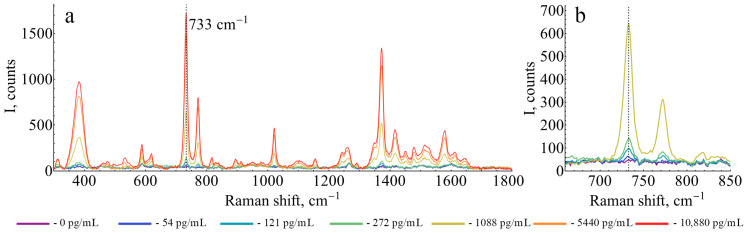
Representative SERS spectra of HRP-catalyzed oxidation product of oPD acquired in SERS-based GM ELISA. Enzymatic reaction was performed at 1 mM oPD and 80 µM H_2_O_2_ in 100 mM citrate buffer at pH 6 with 5 µg/mL of BSA and stopped with 1:2 dilution with 1.5 M citrate buffer pH 3. (**a**) Full SERS spectra at different concentrations of GM (0, 54, 121, 272, 1088, 5440 and 10,880 pg/mL). The polynomial baseline was subtracted from each spectrum. (**b**) Fragment of the same SERS spectra around the analytical band (733 cm^−1^) at lower galactomannan concentration range (0, 54, 121, 272 and 1088 pg/mL).

Next, spiked samples of GM (0, 16, 24, 36, 54, 81, 121, 181, 272, 544, 1088, 5440, and 10,880 pg/mL) in the GM-depleted supernatant of denatured plasma were prepared. Representative SERS spectra of oPD oxidation product in the presence of HRP, obtained during ELISA at different concentrations of GM, are shown in Figure 1.

**Figure 2:**
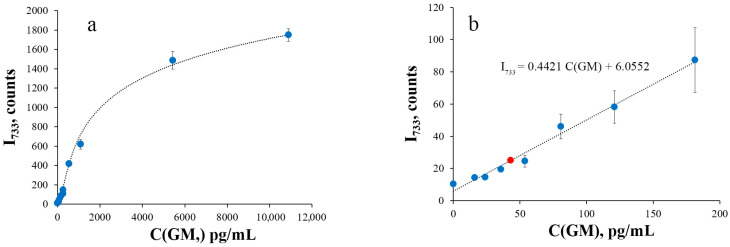
SERS intensity at 733 cm^−1^ of different galactomannan concentrations (N = 9 technical replicates). The enzymatic reaction was performed at 1 mM oPD and 80 µM H_2_O_2_ in 100 mM citrate buffer at pH 6 with 5 µg/mL of BSA and stopped with 1:2 dilution with 1.5 M citrate buffer pH 3. (**a**) Full calibration curve at galactomannan concentrations 0, 16, 24, 36, 54, 81, 121, 181, 272, 544, 1088, 5440, and 10,880 pg/mL. (**b**) Low concentration part of the calibration curve. Red marker indicates LOD. Dashed lines represent calibration curves.

The resulting SERS spectra were processed to obtain the calibration curve for GM measurement (Figure 2). The estimated LOD for the SERS measurement of GM was 43 pg/mL (4.3 pg per assay). The mean CV for the range above the LOD was 11%, which is comparable to the CV previously observed for the measurement of soluble HRP (13%) using the same SERS protocol [32].

**Figure 3:**
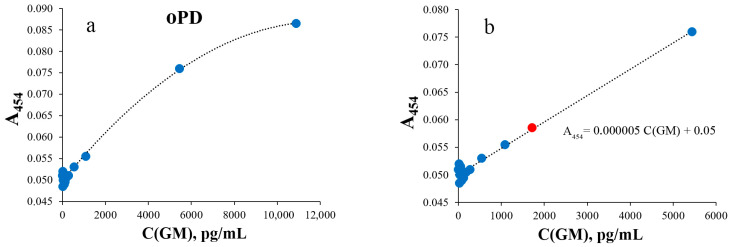
Colorimetric detection of different galactomannan concentrations with oPD substrate (N = 9 technical replicates). Enzymatic reaction was performed and stopped identically to SERS-based readout. Absorbance was measured at 454 nm. (**a**) Calibration curve for different galactomannan concentrations (0, 16, 24, 36, 54, 81, 121, 181, 272, 544, 1088, 5440 and 10,880 pg/mL). (**b**) Low-concentration part of the calibration curve. A red marker indicates LOD. Dashed lines represent calibration curves.

**Figure 4:**
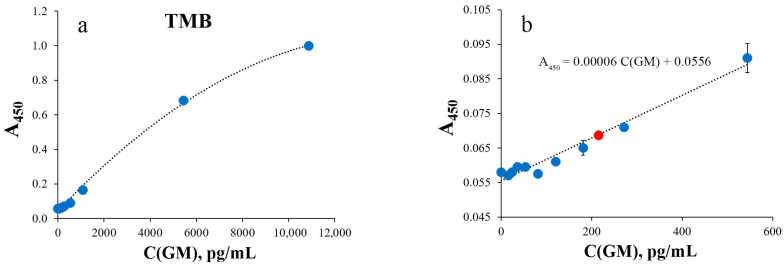
Colorimetric detection of different galactomannan concentrations with TMB (N = 9 technical replicates). Absorbance was measured at 450 nm. (**a**) Calibration curve for different galactomannan concentrations (0, 16, 24, 36, 54, 81, 121, 181, 272, 544, 1088, 5440 and 10,880 pg/mL). (**b**) Low-concentration part of the calibration curve. A red marker indicates LOD. Dashed lines represent calibration curves.

In order to evaluate the performance of the SERS-based GM assay, it was compared to GM colorimetric ELISA. Two options were used. The first one was the same oPD oxidation reaction and the same conditions as for SERS. The second colorimetric readout was performed with a widely used TMB substrate. Both calibration curves (Figure 3 and Figure 4) were linear with a negative deviation at high GM concentrations. The LODs were estimated to be 2000 pg/mL and 216 pg/mL for oPD and TMB as HRP substrates, correspondingly. The mean CVs for the range above the LOD were 3% and 5%, respectively.

Therefore, the SERS detection of GM at 733 cm^−1^ is about one and a half orders of magnitude more sensitive compared to colorimetry under the same conditions (enzymatic reaction at pH 6, 1 mM oPD, and 80 µM H_2_O_2_, stopped by three-fold dilution with 1.5 M citrate buffer at pH 3), and five times more sensitive than the common colorimetric method using TMB as an HRP substrate (Table 1), which confirms the performance of the proposed SERS-based detection system.

The lower difference in the sensitivity of SERS compared to the TMB colorimetric method is explained by the fact that TMB has the highest sensitivity for HRP measurements compared to other chromogenic substrates such as oPD, 2,2-diazo-bis(3-ethylbenzothiazoline-6-sulfonic acid) (ABTS), etc. [44]. In addition, commonly used concentrations of hydrogen peroxide are significantly higher in commercial TMB reagents for ELISA (0.5 to 2.2 mM) than 80 μM, proposed for the colorimetric detection of HRP with oPD [45] and applied for SERS detection [32], which undoubtedly influences the rate of the enzymatic reaction [46,47]. This is also confirmed by the almost tenfold difference in LODs of GM colorimetric assays when using TMB versus oPD as HRP substrates (Figure 3 and Figure 4).

The developed SERS-based ELISA for Aspergillus GM is a promising prototype for commercial assay. SERS readout has been proved to be compatible with sandwich immunochemical procedures in a standard 96-well plate. It retains its previously high sensitivity as shown with a model system of soluble HRP in a real ELISA on plasma samples. We suppose that additional research could be performed to improve the assay’s repeatability (11% for SERS readout vs. 3–5% for colorimetric ones, Table 1). In terms of practical convenience, it could be beneficial to automate SERS readout procedures, such as withdrawal of the aliquot of the oPD-DAP solution after the enzymatic reaction, mixing with stop reagent and AgNPs, followed by SERS spectrum acquisition. Also, as long as SERS is an optical contactless method that requires a small amount of sample (below 20 μL), it is well suited for usage in compact microfluidic devices.

## 4. Conclusions

The current study is the first attempt to combine the previously optimized SERS readout protocol for the HRP–oPD–DAP system using hydroxylamine silver colloids with an ELISA method using highly specific monoclonal antibodies (mAbs) capable of recognizing Aspergillus galactomannan. The presented SERS-based ELISA is approximately one and a half orders of magnitude more sensitive than colorimetry under the same conditions (pH 6, 1 mM oPD and 80 μM H_2_O_2_) and five times more sensitive than the colorimetric method using TMB as the HRP substrate, which confirms the performance of the proposed SERS-based detection system.

## Figures and Tables

**Table 1:** A comparison of the LOD and mean CV values for colorimetric and SERS detection.

Detection Method	Colorimetry		SERS
Substrate	TMB	oPD	oPD
LOD, pg/mL	216	2000	43
CV, %	3	5	11

**Figure A1:**
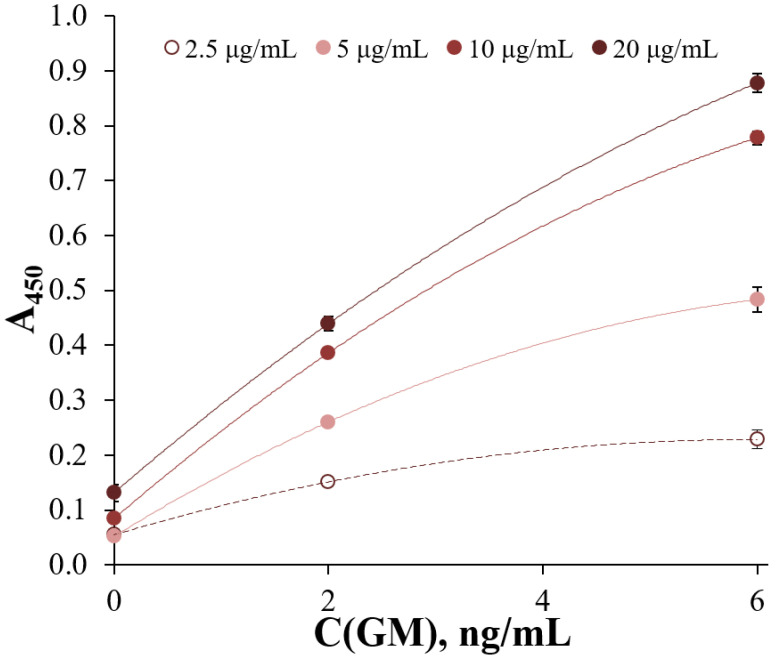
Effect of HRP-conjugated mAb 7B8 concentration on galactomannan calibration curves in TBST matrix.

**Figure A2:**
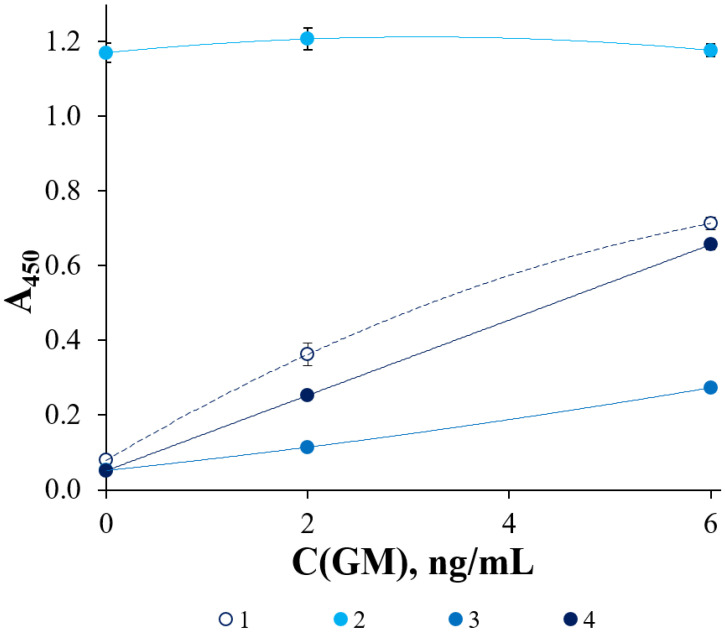
Effect of matrix solution on galactomannan calibration curves: 1—100 mM Tris-HCl, 150 mM NaCl, pH 8 containing 0.05% Tween-20 and 1% BSA; 2—fetal bovine serum; 3—GM-depleted plasma; 4—GM-depleted supernatant of boiled plasma.

## Data Availability

All the relevant data are provided in the main text, Appendix A and Appendix B or Supplementary Materials.

## Supplementary Materials

The following supporting information can be downloaded at: https://www.mdpi.com/article/10.3390/s25144376/s1, Figure S1. UV-visible absorbance spectra of AgNP colloids. (a) All synthesized batches of AgNPs measured in a 15-fold dilution with water. (b) Three selected batches with λ_max_ between 406.6 and 408.6 nm. (c) Around two-fold concentrate of AgNPs transferred into standard medium (20 mM NaCl) and measured in 30-fold dilution with water. (d) Spectra of transferred AgNPs normalized to A_max_ = 0.95.

## Abbreviations

AgNPs	Silver nanoparticles
BALF	Bronchoalveolar lavage fluid
CV	Coefficient of variation
DAP	2,3-Diaminophenazine
EDTA	Ethylenediaminetetraacetic acid
ELISA	Enzyme-linked immunosorbent assay
FBS	Fetal bovine serum
GM	Galactomannan
HRP	Horseradish peroxidase
IA	Invasive aspergillosis
IPA	Invasive pulmonary aspergillosis
LOD	Limit of detection
mAbs	Monoclonal antibodies
ODI	Optical density index
oPD	*o*-Phenylenediamine
SATP	N-succinimidyl S-acetylthiopropionate
SD	Standard deviation
SERS	Surface-enhanced Raman scattering
Sulfo-SMCC	Sulfosuccinimidyl 4-[N-maleimidomethyl] cyclohexane-1-carboxylate
TBST	100 mM Tris-HCl, 150 mM NaCl, pH 8 containing 0.05% Tween-20
TMB	3,3′,5,5′-Tetramethylbenzidine

## Appendix A

### Reagents Used for Buffers and Stop Solutions Preparations

NaH_2_PO_4_ (#71496, ≥99%), Na_2_HPO_4_ (#1.06586, ≥99%), NaCl (#S9625, ≥99%), sodium citrate tribasic dihydrate (#S4641, ≥99%), Tween-20 (#P1379), and bovine serum albumin (BSA, #A7030, ≥98%) were purchased from Sigma-Aldrich, (Burlington, MA, USA). Tris(hydroxymethyl)-aminomethane (TRIS, #A2264, >99.9%) was purchased from PanReac AppliChem,(Monza, Italy). Sulfuric acid (extra pure grade) was purchased from Chimmed, (Moscow, Russia). HCl (extra pure grade, 35–38 weight %), and citric acid monohydrate (extra pure grade) were purchased from Component-Reaktiv, (Moscow, Russia). All solutions were prepared using deionized water (18.2 MΩ·cm) from the MilliQ UF Plus system (Millipore, Molsheim, France).

## Appendix B

### Selection of Optimal Conditions for ELISA

In order to fully exploit the capability of SERS-based HRP readout in ELISA, it is highly important to reduce the amount of HRP in blank samples to the lowest possible level. The two main reasons for high background are non-specific conjugate sorption and the presence of minor GM quantities in the plasma of healthy donors. The current appendix is devoted to solution of these two problems.

First, the optimal concentration of HRP-conjugated mAb 7B8 to minimize the background while keeping the high specific signal was selected. The concentration of the HRP-conjugated mAb 7B8 varied from 2.5 to 20 μg/mL (Figure A1). The ELISA protocol is described below.

The wells of a 96-well clear polystyrene high bind stripwell microplate (# 2592, Corning, NY, USA) were filled with mAb 7B8 (100 μL/well of a 10 μg/mL solution in 100 mM Tris-HCl, 150 mM NaCl, pH 8) and incubated for 16 h at 4 °C. After washing three times with TBST, the plate was blocked by 1% BSA solution in TBST (16 h at 4 °C). After blocking, 100 μL HRP-conjugated mAb 7B8 (2.5, 5, 10 or 20 µg/mL in TBST) and 100 μL of GM-spiked TBST solution were added into the wells and incubated for 60 min at 37 °C. After washing five times, the enzymatic reaction was carried out using 100 μL TMB for 15 min at 37 °C with 600 rpm stirring. Enzymatic oxidation of TMB was stopped with 100 μL of 0.5 M sulfuric acid. Absorbance was measured at 450 nm using xMark plate reader (BioRad, USA). Results were represented as means ± SD.

When the detection antibody HRP conjugate concentration was increased from 2.5 to 5 and further to 10 μg/mL, a substantial improvement in the specific signal was observed (Figure A1). However, the mAb-HRP concentration of 20 μg/mL demonstrates almost equal shift in the signal for C(GM) = 0 and 2 ng/mL, which indicates little (if any) improvement in specific signal but a strong increase in non-specific conjugate sorption. The latter is an adverse effect to be avoided, as stated before. Thus, 10 μg/mL of HRP-conjugated mAb 7B8 could be considered as optimal.

The next step was the selection of a matrix solution for spiked standard samples preparation. Four solutions with different composition were used as a matrix for spiked samples preparation:

(1) 100 mM Tris-HCl, 150 mM NaCl, pH 8 containing 0.05% Tween-20 (TBST) and 1% BSA;
(2) Fetal bovine serum (F9665, Sigma Aldrich);
(3) GM-depleted plasma, prepared by the treatment of healthy donors’ plasma with an excess (1 mg/mL) of magnetic beads modified with mAb 7B8 for 4 h at 4 °C with stirring. Captured native GM was removed with magnetic beads by the external magnetic field;
(4) GM-depleted supernatant of boiled plasma. The plasma of healthy donors was pre-treated with 0.1 M EDTA and boiled for 3 min to dissociate complexes of polysaccharide with serum components and denature proteins. The mixture was centrifuged 20 min at 14,000× *g*, and the pellet was discarded. The supernatant was treated with magnetic beads carrying mAb 7B8 as described before in order to deplete the native GM.

Known stock concentrations of GM in TBST with 1% BSA were spiked into solutions 1–4 in a ratio 1:9. The ELISA was performed as described before with the selected mAb-HRP concentration of 10 μg/mL (Figure A2).

The GM-depleted plasma (matrix 3) showed the lowest specific signal. We attribute this result to the presence of anti-GM antibodies in the pooled healthy donors’ plasma, which bind some of the added GM. The signals in TBST with 1% BSA (matrix 1) and GM-depleted supernatant of boiled plasma (matrix 4) were comparable. However, the blank signal was noticeably lower for matrix 4 due to suppressed non-specific mAb-HRP sorption. The low-molecular components of plasma in matrix 4 likely provide additional blocking of the wells, preventing an undesirable nonspecific signal from the adsorbed antibody-HRP conjugate. Thus, GM-depleted supernatant of boiled plasma is the best matrix for spiked standard samples preparation.

It is worth noting an extremely high level of background signal was observed with GM spikes in FBS. One of the probable explanations is the presence of galactomannan in bovine serum. To the best of our knowledge, there are not any specific studies devoted to the measurement of GM in FBS. However, high levels of GM (∼80 ng/mL) could be detected in the serum of cows with spontaneous acquired aspergillosis or in the serum of calves experimentally infected with *A. fumigatus* [48].

Fungi of the Aspergillus genus are widely distributed in the environment (e.g., soil, air, and plant surfaces), found indoors (e.g., building surfaces, air, and household appliances), and present in drinking water and dust. *A. fumigatus* is the most frequently detected thermophilic fungus in dairy cattle feed [49]. The Aspergillus genus (mainly *A. terreus* and *A. fumigatus*) has also been identified in the digestive tract of dairy cattle and was implicated in degrading plant cell walls [50]. In cows and other ruminants, aspergillosis may be asymptomatic or present in a bronchopulmonary form. It can also cause mastitis, placentitis, and abortion [51]. Additionally, FBS can become contaminated during production.

Although FBS is heated to 56 °C for 30 min during production [52,53] and filtered through 0.1-μm sterile filters to remove potential bacterial and fungal contamination [54], none of these procedures remove free galactomannan from serum.

Adding FBS to medium is known to stimulate the growth of Aspergillus [55]. Although this study focused on the possible protein components of the stimulating effect of FBS, the fact that molecules larger than 10 kDa exert the greatest effect (and the molecular weight of GM can range from 20 to 200 kDa) may indirectly indicate a contribution of this polysaccharide to the nutrient medium for cell wall growth. This polysaccharide is hydrolyzed by Aspergillus β-mannanase.
