# Supplementary material for: Increasing the Sensitivity of Aspergillus Galactomannan ELISA Using Silver Nanoparticle-Based Surface-Enhanced Raman Spectroscopy
Source: Sensors (Basel). 2025 Jul 13;25(14):4376. doi: 10.3390/s25144376 (PMC12299639; doi:10.3390/s25144376)
Supplement: Supplementary file 1 [file sensors-25-04376-s001.zip › sensors-3731550-supplementary.pdf]

Supplementary materials

Increasing the Sensitivity of Aspergillus Galactomannan ELISA Using Silver Nanoparticle-Based Surface-Enhanced Raman Spectroscopy

A.D. Vasilyeva, L.V. Yurina, E.G. Evtushenko, E.S. Gavrilina, V.B. Krylov, N.E. Nifantiev, I.N. Kurochkin

In the procedures of SERS-based measurement of HRP label the current paper fully relies on the results of our previous study [32].

S1. Synthesis and standardization of AgNPs

As long as the feasibility of long-term storage of AgNPs has not been evaluated, the current implementation of SERS-based HRP measurement employs the freshly prepared silver colloids (< 36 hours). In order to prepare them reproducibly, the procedure for AgNPs synthesis and standardization was developed and thoroughly tested [32]. The routine constist of following key steps:

- Synthesis of several AgNP colloids at once using a well-established hydroxylamine method;

- Selection of the AgNP colloid with appropriate mean particle size using the position of absorbance maximum (406.6 - 408.6 nm) in UV-visible spectra;

- Transfer of the selected AgNPs into the standard medium (20 mM NaCl) via double centrifugation;

- Standardization of particle concentration by adjustment to the absorbance at maximum to standard value of 14.25 (0.95 at 15-fold dilution).

This procedure has been shown to result in AgNP colloids with reproducible UV-vis spectra, number-weighted mean hydrodynamic particle size (40±1 nm assessed with nanoparticle tracking analysis, NTA), particle shape (mean aspect ratio of 1.13-1.16 measured by transmission electron microscopy [32,36]), particle concentration ($5.1 \pm 0.8 \times 10^{11}$ particles/mL according to NTA), and colloid medium (20 mM NaCl).

$NH_2OH{\cdot}HCl$ (# H9876, ≥99%), $AgNO_3$ (# 209139, ≥99%), and NaCl (# S9625, ≥99%) were purchased from Sigma-Aldrich, USA. Chemically pure NaOH (>99%) was purchased from Chimmed, Russia. All solutions were prepared using deionized water (18.2 MΩ·cm) from the MilliQ UF Plus system (Millipore, Molsheim, France).

One mL of 10 mM $AgNO_3$ was quickly added to 9 mL of 2.56 mM $NH_2OH{\cdot}HCl$ with 3.33 mM NaOH in a 15 mL polypropylene test tube and immediately stirred using a vortex mixer. Typically, five to seven tubes were prepared at once for further selection. The mixture was stored for 1 h to complete the reaction. Each colloid was characterized with UV–vis absorbance spectrum (300-750 nm) in a 15-fold dilution with water using a Shimadzu UV-1800 spectrophotometer (Figure S1a). To standardize the particle size, samples with a maximum of plasmonic band between 406.6 and 408.6 nm were selected (Figure S1b). Selected AgNP colloids were used for SERS measurements within 36 hours. Prior to SERS experiments, 1.5 mL aliquots of colloid were centrifuged for 10 minutes at 7000 RPM (Heraeus Sepatech Biofuge A). The pellet was resuspended in 1.5 mL of 5 mM NaCl, followed by intensive vortex mixing. Next, the AgNP colloid was subjected to the second centrifugation, followed by resuspension in 0.75 mL of 20 mM

NaCl. Absorbance at maximum was measured (Figure S1c) for this around two-fold concentrate (using a 30-fold dilution in water) and adjusted to A_{max} = 14.25 (0.95×15) by dilution in 20 mM NaCl to standardize the particle concentration. The absorbance spectra after the dilution have not been measured, but the consistency of UV-vis spectra for all used AgNP batches could be confirmed by normalization of data from Figure S1c to A_{max} = 0.95 (Figure S1d). Standardized AgNP colloids were used for SERS within 5 hours to prevent their oxidation with atmospheric oxygen.

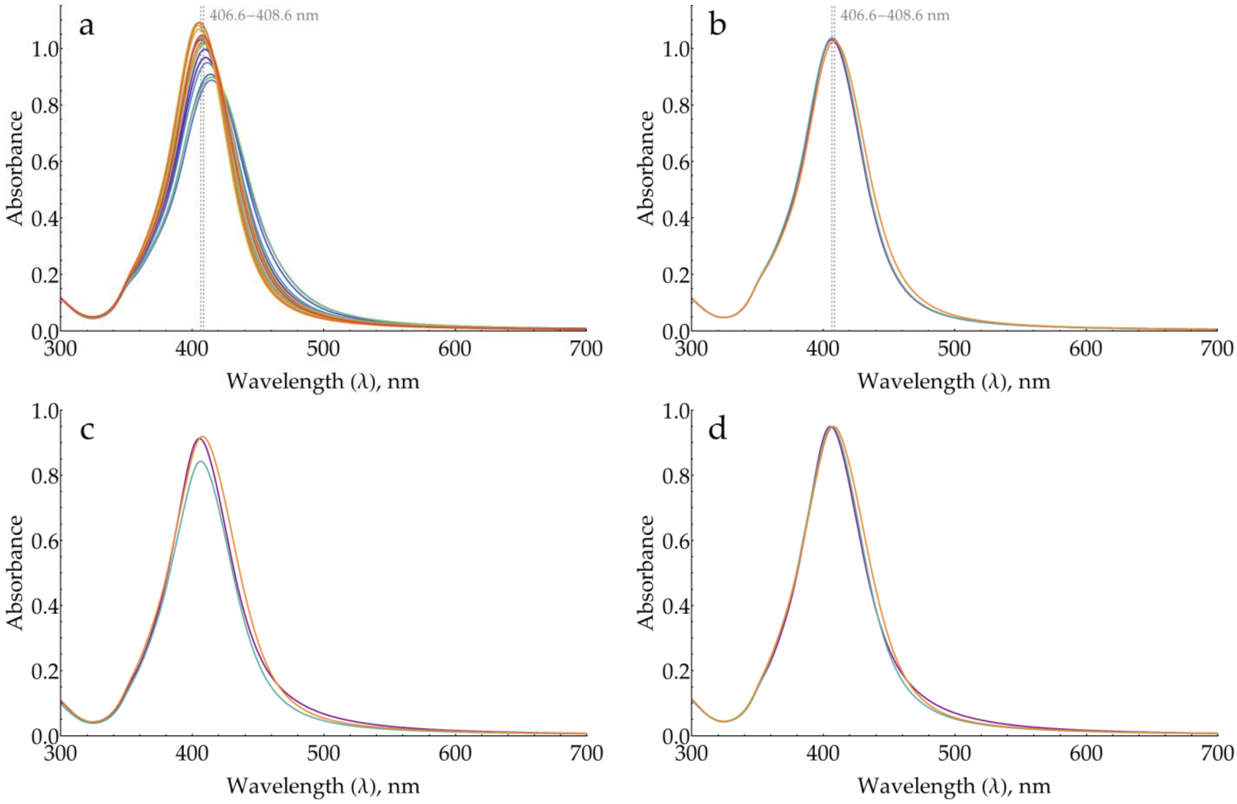

Figure S1. UV-visible absorbance spectra of AgNP colloids. (a) All synthesized batches of AgNPs measured in a 15-fold dilution with water. (b) Three selected batches with λ_{max} between 406.6 and 408.6 nm. (c) Around two-fold concentrate of AgNPs transferred into standard medium (20 mM NaCl) and measured in 30-fold dilution with water. (d) Spectra of transferred AgNPs normalized to A_{max} = 0.95.

S2. Protocol of SERS measurement and spectrum processing

As shown previously [32], the enzymatic reaction mixture stopped by double volume of 1.5 M citrate buffer pH 3 provides the optimal medium for SERS measurement of small amount of enzymatically generated 2,3-diaminophenasine (DAP) in the presence of large excess of unreacted oPD substrate. More specifically, pH 3 reduces the oPD binding to the silver surface and also transforms DAP into its protonated form $DAPH^+$. Citrate buffer diluted down to 1 M provides an optimal aggregation state of AgNPs if SERS spectrum is acquired exactly 2 min after the aggregation initiation.

Thus, 60 μL of AgNPs was added to the stopped reaction mixture (60 μL) with the aggregation timer being started. The sample was thoroughly mixed by pipetting. A droplet of the resulting solution (20 μL) was placed on the surface of thick Al foil. The focus of the spectrometer was positioned at the top surface of the droplet and then shifted 600 μm into the liquid. Two minutes after aggregation had started, the SERS spectrum was acquired. All the data provided in the paper were collected using the portable spectrometer i-Raman Pro BWS475–785 H (BWTek, Plainsboro, NJ, USA) with a 785 nm excitation and 20× objective. The spectrometer setups were as follows: 15 s total signal collection time (0.5 s collection time with 30 automatically averaged repeats) at 135 mW of power on the sample.

To extract quantitative information about the 733 cm^{-1} band intensity from the SERS spectra, 670–805 cm^{-1} range was cut from each spectrum. The non-negative linear superposition of the previously reported peak shapes (see equations in [32], Appendix H), together with the quadratic polynomial background,

was fitted into experimental spectra using the built-in NonlinearModelFit function in Mathematica 10.2 (Wolfram Research, Champaign, IL, USA).
